# Wound infiltration with local anesthetics during cesarean delivery with intrathecal opioids: A systematic review and meta‐analysis of randomized control trials

**DOI:** 10.1002/pmf2.70240

**Published:** 2026-02-06

**Authors:** Valentina Frusone, Kavisha Khanuja, Jacqueline Sinnott, Valentina Bellussi, Guillaume Ducarme, Manuel Gomez‐Rios, Jerome Rosetti, Amanda Roman, Vincenzo Berghella

**Affiliations:** ^1^ Drexel University Philadelphia Pennsylvania USA; ^2^ Division of Maternal‐Fetal Medicine Department of Obstetrics and Gynecology, Thomas Jefferson University Hospital Philadelphia Pennsylvania USA; ^3^ Azienda Ospedaliera Universitaria Integrata AOUI Verona Borgo Trento Verona Italy; ^4^ Department of Obstetrics and Gynecology Centre Hospitalier Departemental La Roche sur Yon France; ^5^ Department of Anesthesiology and Perioperative Medicine Complejo Hospitalario Universitario de A Coruña A Coruña Galicia Spain; ^6^ Department of Obstetrics and Gynecology, Maternal‐Fetal Medicine CHU Brugmann Brussels Belgium

**Keywords:** cesarean delivery, intrathecal opioid, local anesthesia, opioid consumption, pain scores, wound infiltration

## Abstract

**Objective:**

To evaluate the effect on maternal pain of local anesthetic wound infiltration for intra‐cesarean delivery analgesia in patients who also received intrathecal opioids

**Data sources:**

Scopus, PubMed, and Cochrane Central Register of Controlled Trials were searched from the inception of each database to August 2023.

**Study eligibility criteria:**

All randomized controlled trials (RCTs) comparing the use of local anesthetic wound infiltration at the time of cesarean delivery versus no such infiltration in patients who also received intrathecal opioids. The primary outcome was pain scores at 24 h with movement. Secondary outcomes included pain scores at 12, 48, 72 h with movement and at rest, opioid consumption at 48 h, length of hospitalization, and side effects.

**Study appraisal and synthesis methods:**

Results were summarized as mean difference (MD) or risk ratio (RR) with associated 95% confidence intervals. Quality of studies was evaluated by *Cochrane Handbook for Systematic Reviews of Interventions* for judging risk of bias. Heterogeneity was measured using I‐squared (Higgins I^2^).

**Results:**

Eight RCTs (*n* = 762) comparing local anesthetic wound infiltration to no infiltration during cesarean delivery met inclusion criteria. Local anesthetic (usually with ropivacaine, bupivacaine, or levobupivacaine) wound infiltration was associated with significantly lower pain scores with movement compared to no infiltration (MD, −9.07 (−14.62, −3.53) (*I* ^2 ^= 5%) at 48 h. Additional outcomes showed decreased but not statistically different pain scores at rest: 24 h (MD, −2.57 [−7.79, 2.65]), 48 h (−3.98 [MD, −8.18, 0.23]), and 72 h (MD, −4.28 [−9.56, 1.00]). Analysis of morphine consumption equivalents at 48 h showed a significant decrease in the wound infiltration group (MD, −3.09 [−4.46, −1.72], *I* ^2 ^= 46%). Side effects were similar in both groups (nausea, vomiting, pruritus, and sedation).

**Conclusion:**

Single‐dose local anesthetic wound infiltration—of levobupivacaine, ropivacaine, or bupivacaine—administered subfascial at the time of cesarean delivery with intrathecal opioids is associated with reduced postoperative morphine consumption. The pain intensity scores at rest or with movement at 24 h postoperatively are decreased yet not significantly different, but significantly decreased at 48 h with movement and maternal side effects are comparable.

## 1 INTRODUCTION

As of 2023, the global cesarean delivery (CD) rates have increased to 21%, making it the most frequent major surgery performed around the world. In the United States, there are approximately 1 million CD performed annually, and over 25 million are performed globally [1].

In the past, the postoperative CD pain treatment used to primarily involve opioids, which can impact recovery with adverse effects, such as nausea, vomiting, pruritus, sedation, constipation, respiratory depression, and there is a risk of substance use disorder [2]. Enhanced recovery after surgery (ERAS) guidelines regarding CD emphasize the importance of adequate pain treatment for early mobilization, breastfeeding success, and reducing hospital stay [3, 4]. If pain is not properly controlled following CD, it can negatively impact mother–child bonding and increase the patient's development of chronic pain and postpartum depression [5].

Multimodal analgesia is associated with superior pain relief and decreased opioid use with techniques such as high‐volume wound infiltration with local anesthetic being evaluated for intra‐cesarean use [6]. This approach has been used in various procedures and demonstrated to be a safe, simple, and inexpensive option [7]. Reported benefits include improved analgesia, reduced opioid use, reduced side effects, increased patient satisfaction, and reduced length of hospital stay, with noted limitations such as wound catheter displacement, infection risk, cost, and misjudgment of the technique [8]. Various medications such as ropivacaine, bupivacaine, or levobupivacaine can be used in single infiltration or continuous infiltration methods placed either subfascially or suprafacially at incision site. Adding intrathecal lipophilic opioids, such as fentanyl or sufentanil, is highly effective in the management of postoperative pain due to the prolonged analgesia with improved quality of spinal anesthesia and decreases spinal hypotension, decreased postoperative analgesic requirements, and facilitating early mobilization after abdominal surgery [9, 10].

## 2 OBJECTIVE

This systematic review and meta‐analysis of randomized controlled trials (RCTs) aims to evaluate the effectiveness of a multimodal pain approach, specifically of local anesthetic wound infiltration for post‐CD analgesia using either infusion techniques or single wound filtration in patients who also received intrathecal opioids.

## 3 METHODS

### 3.1 Eligibility criteria, information sources, search strategy

The research protocol was developed with defined search methods and inclusion criteria for article selection and subsequent extraction and analysis of data. A list of key words and headings were combined to search the electronic databases PubMed, Scopus, and Cochrane Library from inception through August 2023 and included “cesarean,” “caesarean,” “postoperative,” “analgesia,” “pain,” “wound infiltration,” “intrathecal morphine.” The date of the last search was August 28, 2023. Search strategy was designed with assistance from a research librarian. To locate additional publications, we reviewed bibliographies of identified studies and review articles. No restrictions for language or geographic location were applied. Rayyan was used to screen the abstracts of the articles.

### 3.2 Study selection

We conducted a systematic literature review including all published or unpublished RCTs that evaluated the effectiveness and safety of infiltration of local anesthetics for patients who underwent CD and received intrathecal opioids. We decided to limit our search to individuals that received intrathecal opioids as they are the standard of care currently [3]. Reviews, observational studies, case‐control studies, letters, or case reports were excluded. Inclusion criteria for the participants included pregnant adults at least 18 years of age who underwent CD and various routes of administration such as subcutaneous wound infiltration, infiltration through all layers of the abdomen, or continuous wound infiltration. We excluded participants who were under 18 years of age.

Prior to reviewing articles or data extraction, the study protocol was registered with PROSPERO International Prospective Register of Systematic Review under the identification number CRD42023389042. Titles, abstracts, and full texts were screened by three independent reviewers (V.F., J.S., and K.K.) for inclusion. Any disagreements regarding inclusion were resolved by discussion with a fourth reviewer (V.B.). This systematic review followed the recommendations of the Preferred Reporting Items for Systematic Reviews and MetaAnalyses (PRISMA) [11].

### 3.3 Assessment of risk of bias

Two researchers independently assessed the articles that met the inclusion criteria using the risk of assessment tool using version 2 of the Cochrane RoB tool for randomized trials. The tool assessed five domains: randomization process, deviations from intended interventions, missing outcome data, measurement of the outcome, and selection of the reported result. Each domain was labeled as “high risk of bias” or “low risk of bias” to assign the risk of bias.

**FIGURE 1:**
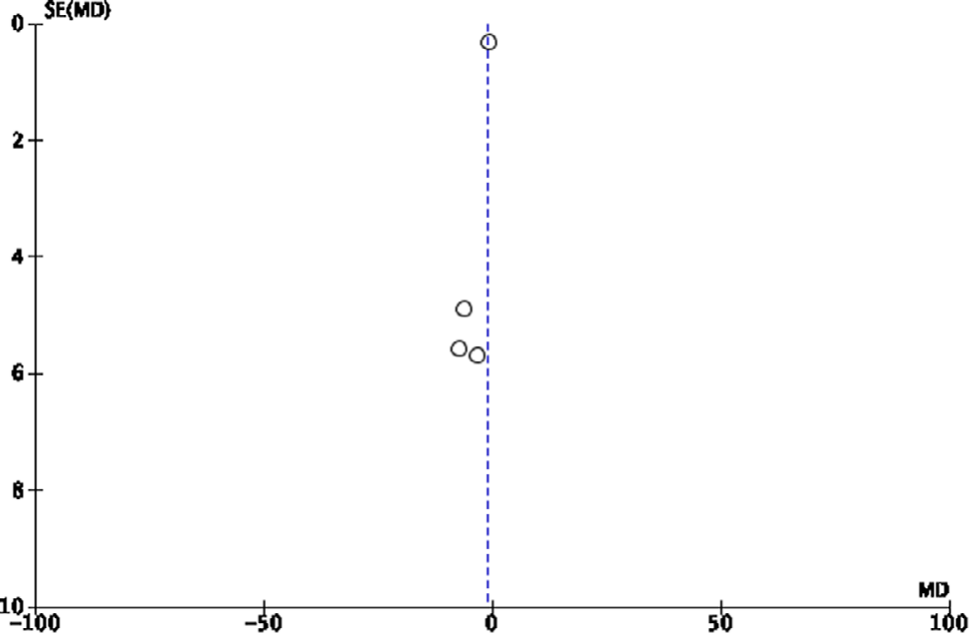
Funnel plot of comparison: pain score with movement at 24 h.

Figure 1, a funnel plot of comparison for the pain score with movement at 24 h, was created for our primary result to assess the publication bias. As demonstrated in our figure, the studies are centered around a mean effect of zero, suggesting minimal publication bias.

### 3.4 Quality of evidence

The Grading of Recommendations Assessment, Development and Evaluation (GRADE) system was used to assess evidence quality and strength of evidence recommendation [12]. An absolute RCT trustworthiness criterion table is included in the Supporting Information.

### 3.5 Data extraction

Data extraction was performed by two independent investigators (V.F. and K.K.), who created a data collection sheet and independently extracted and analyzed data. Each investigator evaluated the full research papers after screening through the abstracts. Independently they wrote the reasoning behind including or excluding a study. When a disagreement arrived, the investigators specified how they came to their own conclusion and reasoned through the final decision, with a third reviewer resolving any conflicts. The primary outcomes for this study were the pain intensity scores at 24 h with patient mobilization (Table 1). The secondary outcomes were pain intensity scores at 12, 48, and 72 h with movement and at rest (Table 1), opioid consumption at 48 h (Table 2), duration of hospitalization, and side effects (Table 3). In the studies that displayed their data by evaluating the median of the sample, an online calculator was used to generate the mean of the sample with the standard deviation [13]. Few studies had data that were too skewed to be converted to the mean with standard deviation. All pain scores were converted to a 0‐to‐100‐point scale. Opiate drugs other than intrathecal morphine were converted to morphine equivalents [14]. Authors were contacted for missing results following data extraction where necessary.

**TABLE 1:** Pain scores of trials reporting.

Study	Numerical scale used	Frequency of testing	Pain score post op at rest					Pain Score post op with movement				
			6	12	24	48	72	6	12	24	48	72
Pavy, 1994 [17]	0–100	Pain scores at rest, pain with movement and worst pain at 24, 48, and 72 h			1.0 (0.0–8.0) (I) 2.4 (0.0–6.5) (C)	15 (0–100) (I) 24 (0–47) (C)	8 (0–100) (I) 17 (0–73) (C)			51 (6–100) (I) 47(0–90) (C)	50 (6–100) (I) 50 (6–89) (C)	32 (8–99) (I) 44 (2–90) (C)
Ducarme, 2012 [18]	0–100	Pain scores with movement every 20 min for 1 h then every 4 h for the first 12 h postpartum, then at the 24th and 48th h							11.9 ± 1.7 (I) 10.2 ± 1.7 (C)	17.5 ± (1.0) (I) 18.4 ± (1.9) (C)		
Niklasson, 2012 [19]	0–100	Pain scores at rest at 6, 12, 24 h		39.6 ± 15.7 (I) 42.7 ± 13.8 (C)	44.9 ± 13.1 (I) 45.4 ± 12.1 (C)							
Gomez Rio, 2022 [24]	0–100	Pain scores at rest and with movement postoperatively at 30 min, 1, 2, 4, 12, 24, 48, and 72 h			4.4 ± 7.2 (I) 9.4 ± 13.7 (C)	5.0 ± 10.8 (I) 6.7 ± 13.7 (C)	0.9 ± 2.9 (I) 5.3 ± 14.6 (C)			34.7 ± 23.7 (I) 41.9 ± 22.7 (C)	22.8 ± 18.4 (I) 26.4 ± 22.2 (C)	17.5 ± 16.1 (I) 19.4 ± 22.5 (C)
Rosetti, 2021 [23]	0–100	Pain scores at rest and with mobilization at 2, 6, and 12 h, once daily after surgery until discharge.	27.3 ± 19.8 (I) 37.9 ± 24.3 (C)	35.3 ± 21.9 (I) 36.7 ± 24.6 (C)	30.0 ± 18.9 (I) 23.3 ± 20.6 (C)	14.6 ± 16.5 (I) 16.4 ± 17.6 (C)	11.9 ± 18.1 (I) 10.5 ± 11.6 (C)	39.0 ± 26.6 (I) 53.6 ± 25.5 (C)	47.8 ± 26.3 (I) 51.0 ± 25.9 (C)	43.1 ± 23.5 (I) 46.4 ± 23.6 (C)	31.4 ± 20.9 (I) 41.3 ± 22.4 (C)	33.1 ± 23.3 (I) 33.3 ± 19.7 (C)
Larsen, 2015 [21]	0–100	Pain scores at incision at rest, from supine to sitting position and during walking immediately after surgery and at 3, 6, 8, 12 and 24 h	20 (4–32) (I 0.2%) 18.6 ± 21.8 20 (2–30) (I 0.5%) 17.1 ± 21.8 16 (2–46) (C) 21.7 ± 34.3	33 (15–58) (I 0.2%) 35.5 ± 33.4 23 (10–42) (I 0.5%) 25.1 ± 24.9 20 (10–45) (C) 25.3 ± 27.3	11 (0–18) (I 0.2%) 9.6 ± 14.0 10 (0–12) (I 0.5%)^a^ 5 (0–10) (C) 5.0 ± 7.8			35 (0–85) (I 0.2%) 41.5 ± 68.5 35 (0–85) (I 0.5%) 40.4 ± 66.3 35 (18–50) (C) 34.3 ± 25.0	51 (28–72) (I 0.2%) 50.3 ± 34.3 57 (38–76) (I 0.5%) 57.0 ± 29.7 47 (25–68) (C) 46.6 ± 33.6	20 (10–38) (I 0.2%) 22.9 ± 21.8 22 (11–52) (I 0.5%) 28.8 ± 31.9 24 (11–50) (C) 28.6 ± 30.5		
Jolly, 2015 [20]	0–100	Pain scores at rest and during lower‐limb mobilization at 2 4, 8, 12, 16, 20, 24, 36, 48, and 72 h		15 ± 15(I) 21 ± 19(C)	9 ± 11(I) 19 ± 19(C)	8 ± 11 (I) 16 ± 18(C)	7 ± 10(I) 16 ± 15(C)		30 ± 17 (I) 38 ± 23(C)	33 ± 18(I) 39 ± 22(C)	25 ± 16(I) 38 ± 20(C)	23 ± 17(I) 32 ± 21(C)
Prabhu, 2018 [22]	11 point numeric scale	Pain scores at rest at 24, 48, and 72 h postoperatively			2 (1–3) (I) 2 (1–4) (C)	2 (1–4) (I) 2 (1–3) (C)	2 (1–3) (I) 2 (0–3) (C)			4 (3–6) (I) 5 (3–6.5) (C)	4 (2–5) (I) 3.5 (2–5.5) (C)	2.5 (2–4) (I) 3 (3–6) (C)
*I* ^2^				9%	72%	19%	58%		46%	0%	5%	0%
RR or MD (95% CI)				−2.76 [−6.39, 0.88]	−1.09 [−5.87, 3.68]	−3.98 [−8.18, 0.23]	−4.28 [−9.56, 1.00]		−1.90 [−7.42, 3.61]	−0.96 [−1.58, −0.34]	−9.07 [−14.62, −3.53]	−3.96 [−9.39, 1.48]

*Note*: Data are reported as number (percentage), mean ± standard deviation, or median (interquartile range).

Abbreviations: C, control; CI, confidence interval; I, Intervention; MD, mean difference; RR, risk ratio.

^a^Skewed data.

**TABLE 2:** Secondary outcomes.

Name, year	Type of opioid	48 h mean opioid consumption local versus without local (morphine milligram equivalents)	Mean length of stay (days)
Pavy, 1994 [17]	Codeine with tylenol	NR	NR
Ducarme, 2012 [18]	IV morphine 3 mg bolus	12 ± 4 (I) 15 ± 3 (C)	5.1 ± 0.6 (I) 5.1 ± 0.5 (C)
Niklasson, 2012 [19]	Morphine	37.5 (7.5–60) (I) 37.5 (15–75) (C)^a^	NR
Jolly, 2015 [20]	Morphine	13.1 (I) 19.8 (C)	NR
Larsen, 2015 [21]	Ketobemidone	24 (12–48) (0.5 I) 36 (24–48) (0.2 I) 24 (24–48) (C)^a^	NR
Prabhu, 2018 [22]	Oxycodone	56.3 (11.3–78.8) (I) 48.3 ± 51.9 56.3 (22.5–90) (C) 56.3 ± 51.9	3.46 ± 1.0 (I) 3.56 ± 0.5 (C)
Rosetti, 2021 [23]	Morphine	21.52 ± 21.56 (I) 29.57 ± 22.38 (C)	NR
Gomez‐Rios, 2022 [24]	Morphine	9 (4–16) (I) 9.7 ± 9.3 18 (9–34) (C) 20.5 ± 19.3	3 (3–4) (I) 3 (3–4) (C)
Total	5/8 morphine 1/8 codeine 1/8 ketobemidone 1/8 oxycodone	*I* ^2^ = 46% −6.05 [−10.92, −1.17]	

*Note*: Data are reported as number (percentage), mean ± standard deviation, or median (interquartile range).

Abbreviations: C, control; CI, confidence interval; I, Intervention; MD, mean difference; NR, not applicable.

^a^ Skewed data.

**TABLE 3:** Maternal side effects within 48 h.

Name, year	Nausea, *N*/total (%)	Vomiting, *n*/total (%)	Pruritus, *N*/total (%)	Sedation, *N*/total (%)
Pavy, 1994 [17]	1/20 (5) (I) 7/20 (35) (C)	NR	5/20 (25) (I) 9/20 (45) (C)	NR
Ducarme, 2012 [18]	2/44 (4.5) (I) 3/56 (5.3) (C)	2/44 (4.5) (I) 3/56 (5.3) (C)	0 (0) (I) 0 (0) (C)	0 (0) (I) 0 (0) (C)
Niklasson, 2012 [19]	NR	NR	NR	NR
Jolly, 2015 [20]	NR	3 (8.8%) (I) 3 (8.8%) (C)	NR	NR
Larsen, 2015 [21]	NR	NR	NR	NR
Prabhu, 2018 [22]	13/39 (33.3) (I) 18/40 (45.0) (C)	NR	30/39 (76.9) (I) 34/40 (85.0) (C)	NR
Rosetti, 2021 [23]	2 (5.71) (I) 1 (2.94) (C)	NR	2 (5.71) (I) 1 (2.94) (C)	0 (0) (I) 0 (0) (C)
Gomez‐Rios, 2022 [24]	1(3.0) (I) 1(2.7) (C)	0(0) (I) 0(0) (C)	0(0) (I) 0(0) (C)	0(0) (I) 0(0) (C)
Total	10.3% (I) 18.1 % (C)	4.3% (I) 4.7% (C)	21.5% (I) 26.6 % (C)	0% (I) 0% (C)
RR	0.72 (0.44–1.18)	0.92 (0.27–3.18)	0.60 (0.27, 1.33)	NR
*p*	0.49	0.89	0.54	NR

*Note*: Data are reported as number (percentage), mean ± standard deviation, or median (interquartile range).

Abbreviations: C, control; CI, confidence interval; I, Intervention; MD, mean difference; NR, not applicable; RR, risk ratio.

### 3.6 Data synthesis

Data analysis was performed independently by two authors (V.F. and K.K.) using Review Manager 5.4.1 (The Nordic Cochrane Centre, Cochrane Collaboration, 2014) [15].

Risk ratios (RRs) with 95% confidence interval (CI) compared binary outcomes, whereas mean differences with 95% CI compared continuous outcomes between patients with and without local wound infiltration.  Completed analyses were compared and differences were resolved by reviewing the data set and the independent analyses.

Meta‐analysis was performed using the random effects model of DerSimonian and Laird to produce summary treatment effects [16]. Heterogeneity was measured using Higgins *I*‐squared (*I* ^2^). A *p* value < 0.05 was considered statistically significant.

## 4 RESULTS

### 4.1 Study selection

**FIGURE 2:**
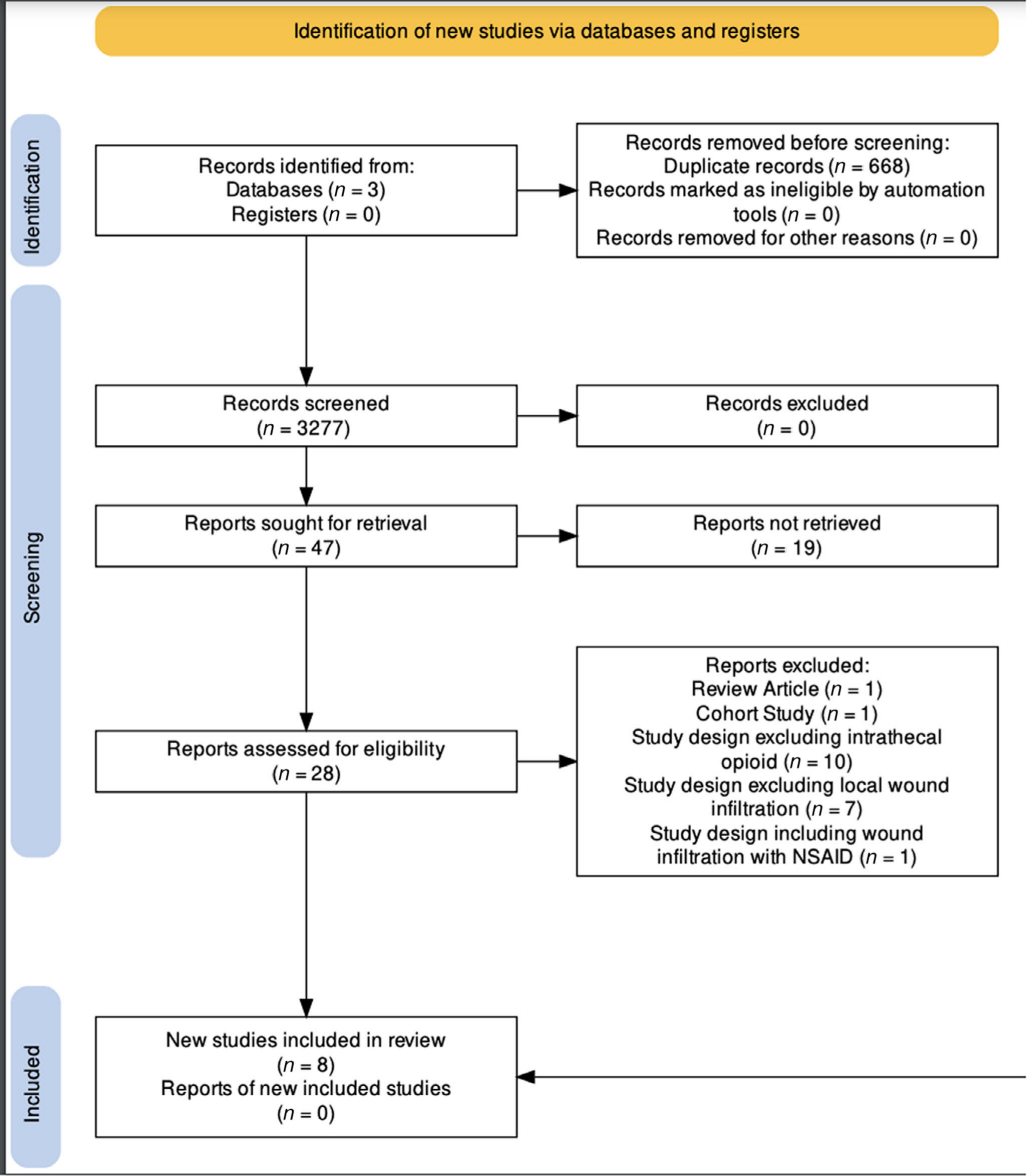
PRISMA flow chart of literature search. PRISMA, Preferred reporting Items for Systematic Reviews and MetaAnalyses.

Our systematic search identified a total of 3277 unique records screened by title and abstract. Forty‐seven articles were considered potentially eligible. In total, eight studies met all inclusion criteria for this study (PRISMA diagram, Figure 2) [17, 18, 19, 20, 21, 22, 23, 24]. All authors were contacted with a request for supplemental data and these data were received from authors of three of the eight studies [18, 23, 24].

### 4.2 Study characteristics

The baseline characteristics of the eight studies included in our systematic review and meta‐analysis are summarized in Table 4. Among all eight studies, there were a total of 762 patients with 402 randomized to wound infiltration and 360 randomized to standard of care. All studies enrolled patients prior to delivery. Most studies only included patients undergoing planned CD [17, 18, 19, 20, 21, 22, 24], whereas only one study included all patients undergoing any type of CD [23]. Inclusion and exclusion criteria varied by study.

**TABLE 4:** Characteristics of included trials.

Name, year	Study location	Number of participants (intervention/control)	Inclusion criteria	Exclusion criteria	Primary outcome	Study duration (h)
Pavy, 1994 [17]	Canada	40 (20/20)	English speakers, ASA 1–2 undergoing scheduled elective C/D	Non‐English speaking	Pain scores at rest, with movement, and worst pain in last 24 h	72
Ducarme, 2012 [18]	France	100 (55/44)	All scheduled C/D under spinal anesthesia	Contraindication to locoregional anesthesia or ropivacaine	Quantification of pain using VAS score at cesarean skin incision on cough and leg raise during the first 48 postoperative h after CD	48
Niklasson, 2012 [19]	Sweden	249 (127/122)	Healthy, 18–50 years old women, for scheduled C/D after 38w0d gestation, Swedish speaking	Ongoing treatment for chronic pain, history of narcotic abuse, severe psychiatric history and any intolerance to opioids, local anesthetics or other analgesic drugs given in the study.	Morphine consumption and mean resting pain intensity at 12 and 24 h	24
Jolly, 2015 [20]	France	68 (34/34)	Aged 18–50 years; full term (37–42 weeks), scheduled elective C/D under spinal anesthesia	Emergency C‐Section; contraindication to morphine, acetaminophen, or local anesthetics; coagulation abnormalities, active infection, insulin‐ treated diabetes; history of opioid use for more than 6 months; insufficient comprehension of French.	Morphine consumption by PCA over the first day after cesarean section.	72
Larsen, 2015 [21]	Denmark	87 (29/30/28)	All patients scheduled for C/D	Alcohol and drug abuse, daily treatment with opioids or glucocorticoids, allergy to ropivacaine or opioids, under the age of 18, breastfeeding, psychiatric disease and non‐Danish language.	Postoperative pain at rest, from supine to sitting position, and with walking immediately after surgery and at 3, 6, 8, 12, and 24 h after operation.	24
Prabhu, 2018 [22]	United States	79 (39/40)	18 years of age, undergoing scheduled C/D via Pfannenstiel incision with neuraxial anesthesia	Current or prior opioid use ever, allergy to local anesthetic	Pain score with movement at 48 h postop	72
Rosetti, 2021 [23]	Belgium	69 (35/34)	18 years of age, gestational age 34 weeks and greater, ASA Class I, II, undergoing all C/D	Allergies to anesthesia, ASA III or higher, preeclampsia or eclampsia, PGDM, PPH, BMI > 35, preoperative opioid consumption, psychiatric disorders, patient refusal to participate, and language barrier.	Postoperative morphine consumption in the first 48 h post op. Pain intensity evaluated with the VAS at rest and at mobilization at 2, 6, 12 h after surgery and once daily until discharge was a secondary outcome.	48
Gomez‐Rios, 2022 [24]	Spain	70 (33/37)	Women of Teresa Herrera Hospital undergoing scheduled C/D under spinal anesthesia, ASA I or II, age 18–45	ASA status III or higher, refusal to participate, inability to consent, history of clinically significant cardiovascular, pulmonary, hepatic, renal, neurologic, psychiatric, or metabolic disease, PreE, HELLP, coagulopathy; PPH; allergy to study drugs; preexisting infection; previous treatment with opioids or antidepressants; chronic pain; alcohol or drug abuse; consumption of medications that interfere with levobupivacaine metabolism; and more than one previous C/D.	Peri‐incisional area of mechanical secondary hyperalgesia at 24, 48, and 72 h.	72
Total					3/8 studies evaluated the primary outcome until 48 h post operation. 5/8 studies evaluated the primary outcome until 24 h post operation.	

Abbreviations: ASA, American Society of Anesthesiologist class; BMI, body mass index; C/D, cesarean delivery; HELLP, hemolysis, elevated liver enzyme levels, and low platelet; PCA, patient controlled anesthesia; PGDM, pregestational diabetes mellitus; PPH, postpartum hemorrhage; PreE, preeclampsia; VAS, visual analog scale.

Patients included in each trial were of similar age, body mass index, and gestational age (Table 5). Of the studies included, five used single wound infiltration for delivery of local anesthetic while three studies used continuous wound infiltration at the site of incision. The location of infiltration for one study was noted as “skin incision site” and did not inform relation to fascial layer (Table 6).

**TABLE 5:** Characteristics of included women.

Name, year	Maternal age (mean ± SD)	Gestational age at randomization	Gestational age range (weeks)	BMI (kg/m^2^)
Pavy 1994 [17]	33.8 ± 3.6 (I) 31 ± 5.2 (C)	NR	NR	29.4 ± 3.12 (I) 29.0 ± 3.0 (C)
Ducarme 2012 [18]	32.0 ± 5.6 (I) 32.5 ± 5.5 (C)	39.3 ± 0.9 (I) 39.2 ± 0.8 (C)	38–40	30.9 ± 4.3 (I) 30.1 ± 4.7 (C)
Niklasson, 2012 [19]	35 (24–29) (I) 34 (21–48) (C)	NR	Cesarean section planned from 38 full weeks	29.3 (20.3–45.3) (I) 29.2 (22.5–40.7) (C)
Jolly, 2015 [20]	31.5 (21–42) (I) 32 (21–42) (C)	NR	37–42	29.5 ± 4.8 (I) 30.8 ± 5.1 (C)
Larsen, 2015 [21]	33 (35.75–35.25) (C) 32 (30–34) (0.2 I) 31 (27–34) (0.5 I)	NR	NR	NR
Prabhu, 2018 [22]	35.6 ± 4.2 (I) 34.7 ± 3.7 (C)	39 (39–39) (I) 39 (39–39) (C)	39	26.2 ± 5.3 (I) 27.6 ± 6.4 (C)
Rosetti, 2021 [23]	32 (I) 34 (C)	38.43 ± 1.81(I) 38.71 ± 1.32 (C)	>34	29.64 ± 3.50 (I) 27.88 ± 3.99 (C)
Gomez–Rios, 2022 [24]	33 (30–36) (I) 35 (33–40) (C)	39 (38.6–39.6) (I) 39 (38.4–39.8) (C)	38–40	29 (26.8–31.2) (I) 29.3 (26.6–33) (C)
Mean/range	33.8 ± 4.8 (I) 32.7 ± 4.9(C)		34–42	29.12 ± 4.38 (I) 29.08 ± 4.96(C)

*Note*: Data are reported as mean (interquartile range) or ± standard deviation in the intervention versus control group.

Abbreviations: C, control; CI, confidence interval; I, Intervention; MD, mean difference.

**TABLE 6:** Preoperative and Intraoperative pain management.

Name, year	Intervention group	Method of infiltration/ timing of intervention	Location of infiltration	Control group	Intrathecal opioids	Intrathecal anesthetic	Acetaminophen	Other
Pavy, 1994 [17]	20–30 mL 0.5% bupivacaine	Single infiltration before incision	Skin Incision Site	20–30 mL 0.9% saline	250–300 µg morphine + 10 µg fentanyl	0.75% bupivacaine	NR	NR
Ducarme, 2012 [18]	20 cc of 7.5 mg/mL ropivacaine	Single infiltration at wound closure	Supra‐ and subfascial	20 mL of 0.9% saline	100 µg morphine + 2.5 µg sufentanil	10 mg bupivacaine	NR	NR
Niklasson, 2012 [19]	40 mL bupivacaine (2.5 mg/mL) with adrenaline (5 ug/mL)	Single infiltration at wound closure	Fascia and the subcutaneous fat above fascia	40 mL 0.9% saline	0.3 mL fentanyl	1.8–2.6 mL bupivacaine	1 g IV	NR
Jolly, 2015 [20]	Levobupivacaine infusion with 20 cc of 2.5 mg/mL bolus followed by 1.25 mg/mL at a rate of 5 mL/h for 48 h	Wound infiltration catheter with initial bolus at skin closure followed by continuous infusion	Subfascial placement of catheter	NR	5 µg of sufentanil	10 mg bupivacaine	1 g IV	20 mg IV nefopam
Larsen, 2015 [21]	30 cc ropivacaine 0.5% (0.5 I) 125 mL ropivacaine 0.2% (0.2 I)	Single infiltration at wound closure	Deep and superficial muscle, subcutaneous layer	50 mL 0.9% saline	2.5 ug sufentanil	10 mg 0.5% bupivacaine	NR	Cefuroxime 1.5 g IV for infectious prophylaxis
Prabhu, 2018 [22]	80 cc liposomal bupivacaine	Single infiltration at wound closure	Subfascial and subdermal spaces	80 mL 0.9% saline	morphine	Unspecified	NR	NR
Rosetti, 2021 [23]	5 mL ropivacaine 0.2% bolus followed by 7 mL/h infusion for 48 h	Wound infiltration catheter with initial bolus at skin closure followed by continuous infusion	Subfascial placement of catheter, above closed parietal peritoneum	5 mL 0.9% saline 0.9% bolus followed by infusion at 7 mL/h	2–2.5 µg sufentanil	8–10 mg 0.5% bupivacaine	NR	NR
Gomez‐Rios, 2022 [24]	0.35% levobupivacaine followed by 7 mL/h infusion for 48 h	Wound infiltration catheter with initial bolus at skin closure and continuous infusion	Subfascial placement of catheter, above the closed rectus muscles	0.9% saline	10 µg fentanyl	0.5% bupivacaine	NR	NR
Totals	3/8 ropivacaine, 3/8 bupivacaine, 2/8 levobupivacaine	5/8 single infiltration. 3/8 Continuous wound infiltration	6/8 subfascial space 3/8 suprafascial space 1/8 skin incision	7/8 0.9% saline, 1/8 NR	4/8 sufentanil 3/8 fentanyl 3/8 morphine	7/8 bupivacaine, 1/8 unspecified	2/8 acetaminophen use	1/8 nefopam

Abbreviations: IV, intravenous; NR, Not applicable.

The majority of participants in all studies were undergoing a repeat CD, with Pfannenstiel skin entry as the most common incision. All patients received an intrathecal opioid at the time of surgery. Postoperatively, participants in six studies received both acetaminophen and nonsteroidal anti‐inflammatory drug (NSAID) whereas in one study participants only received NSAID (Table 7).

**TABLE 7:** Immediate postoperative medications used in both groups.

Name, Year	Postoperative pain meds—standing	Postoperative pain meds—as needed	PCA	Anti‐emetics, anti‐itch
Pavy, 1994 [17]	NR	30 mg codeine phosphate q 3 h as needed 325 mg acetaminophen q 3 h	NR	NR
Ducarme, 2012 [18]	1 g acetaminophen q 6 h 50 mg ketoprofen q 8 h 20 mg nefopam q 6 h	3 mg IV morphine to achieve VAS score less than 3	NR	Metoclopramide
Niklasson, 2012 [19]	400 mg ibuprofen immediately 200 mg ibuprofen q 6 h 1 g acetaminophen q 6 h	1.0 mg/mL IV morphine until NRS < 3 75 mg codeine q 6 h	NR	30 mL oral paraffin emulsion
Jolly, 2015 [20]	400 mg celecoxib, 1 g oral tylenol, and 20 mg nefopam immediately 200 mg celecoxib BID 1 g tylenol qid 20 mg oral nefopam qid	NR	1.2 mg morphine prn 0.06 mg droperidol boluses prn	4 mg IV betamethasone 0.625 mg IV droperidol
Larsen, 2015 [21]	0.15 µg/kg sufentanil immediately 600 mg ibuprofen 2 g acetaminophen BID	5–10 mg oral ketobemidone if VAS > 5	NR	NR
Prabhu, 2018 [22]	30 mg Ketorolac q6 for 24 h followed by 600 mg ibuprofen q 6 h 650 mg tylenol q 6 h	5–10 mg oxycodone q 4 h 650 mg tylenol q 6 h 600 mg ibuprofen q 6 h	NR	NR
Rosetti, 2021 [23]	75 mg IV diclofenac and 1 g IV Tylenol q 6 h immediately 75 mg IV Diclofenac q 12 h	NR	2 mg morphine with max dose of 20 mg per 4 h	Metoclopramide, ondansetron, subcutaneous naloxone for pruritus
Gomez‐Rios, 2022 [24]	50 mg IV dexketoprofen immediately and 50 mg IV dexketoprofen q 8 h	1 g acetaminophen q 6 h if VAS ≥3 1 mg morphine if VAS ≥3 with max hourly dose of 6 mg	1 mg morphine if VAS ≥3 with max hourly dose of 6 mg	IV ondansetron.
Totals			3/8 morphine PCA	1/8 metoclopramide, 1/8 betamethasone and droperidol 1/8 used oral paraffin emulsion 1/8 used Metoclopramide, ondansetron and subcutaneous naloxone, 1/8 used ondansetron

Abbreviations: bid, twice a day; IV, intravenous; NR, not applicable; NRS, numerical rating scale; PCA, patient controlled anesthesia; q, every; qid, four times a day; VAS, visual analog scale.

### 4.3 Risk of bias of included studies

**FIGURE 3:**
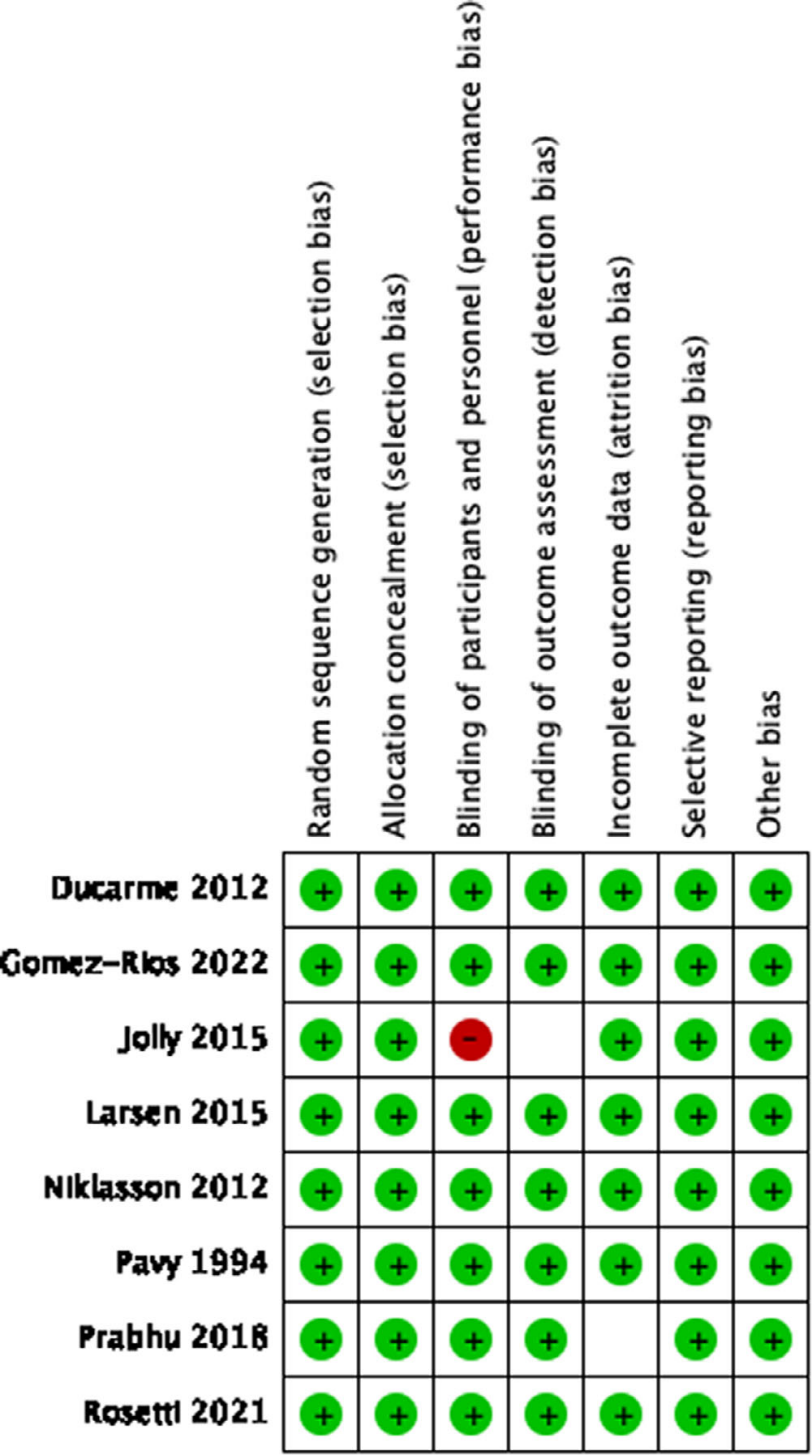
Risk of bias assessment.

Risk of bias of included studies are described in Figure 3. The risk of performance bias was low in all studies, except for one where neither participants nor surgeons were blinded to treatment. There were adequate methods for allocation of subjects and random sequence generation for all studies. Most studies include a generally low risk of bias in blinding of outcome, incomplete outcome data, and selective reporting.

### 4.4 Synthesis of results

**FIGURE 4:**
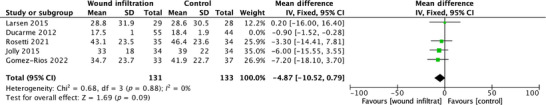
Forest plot for pain scores (0 to 100 scale) at 24 h with movement. CI, confidence interval; IV, inverse variance; SD, standard deviation.

**FIGURE 5:**
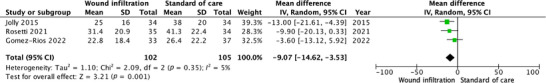
Forest plot for pain scores (0 to 100 scale) at 48 h with movement. CI, confidence interval; IV, inverse variance; SD, standard deviation.

Seven studies reported on our primary outcome of pain intensity score with movement at 24 h postoperatively; however, for our analysis of the primary outcome, five of the studies were included (*n* = 393) and two excluded as data were considered too skewed for conversion to mean and standard for application in meta‐analysis (Table 1). Patients who received wound infiltration at the time of CD reported a lower pain score with movement at 24 h compared to the standard of care group (MD, −0.96 [−1.58, −0.34], *p* = 0.002) with low heterogeneity noted (*I* ^2 ^= 0%). Upon further analysis of results, Ducarme et al. skewed meta‐analysis results left given significant weight (99%) [18]. Therefore, a leave one out sensitivity analysis was performed and demonstrated a trend toward lower reported pain scores, but results were not significant (MD, −4.87 [−10.52, 0.79], *p* = 0.09) (Figure 4). However, patients with anesthetic wound infiltration were noted to have a significantly lower reported pain score with movement at 48 h postoperatively (MD, −9.07 [−14.62, −3.53], *p* = 0.001) (Figure 5).

Seven studies reported on pain intensity score at rest, with five studies included for analysis and two excluded as data were considered too skewed for conversion to mean and standard for application in meta‐analysis. No difference was found in pain scores at rest between patient receiving local wound infiltration and standard of care at different time points, with a non‐significant decrease noted for each: 12 h (MD, −2.76 [−6.39, 0.88]), 24 h (MD, −1.09 [−5.87, 3.68]), 48 h (3.98 [MD, −8.18, 0.23]), and 72 h (MD, −4.28 [−9.56, 1.00]). Seven studies reported on secondary outcome of mean opioid consumption at 48 h, with four studies (*n* = 317) included for analysis and three excluded as data were considered too skewed for conversion to mean and standard for application in meta‐analysis. Patients who received wound infiltration at CD had a significant decrease in mean opioid consumption (defined in morphine milligram equivalents) compared to standard of care (MD, −6.05 [−10.92, −1.17], *p* ≤ 0.00001), with low to moderate heterogeneity noted (*I* ^2 ^= 46%).

Only five studies reported on maternal side effects including nausea, vomiting, pruritus, and sedation, with similar outcomes noted in patients receiving wound infiltration and standard of care. One study reported that five women had their dressings changed due to leakage [19]. Another study stated that wound dressing changes were required for more women in the infiltration group, but no woman had more than one dressing change during her stay [20]. Prabhu et al.’s study showed that in the placebo group, one patient had a wound seroma and two had a wound infection or cellulitis, and in the experimental group one patient noted wound infection or cellulitis [22].

## 5 COMMENT

### 5.1 Principal findings

Our meta‐analysis of RCTs evaluating the use of local wound infiltration at time of CD in patients receiving intrathecal opioid showed a significant decrease in pain intensity score up to 48 h with movement. Use of wound infiltration was also associated with a significant reduction in mean morphine consumption at 48 h compared to standard of care. Importantly, there was no difference in maternal side effects such as nausea, vomiting, pruritus, and sedation.

### 5.2 Results in the context of what is known

CD is associated with moderate to severe postpartum pain with almost one in five women experiencing severe pain [25]. Studies have suggested that the severity of pain during the immediate postpartum period is associated with the development of chronic pain and perinatal mood disorders of pregnancy [5]. Pain after CD is often under‐treated given concerns for both maternal and neonatal side effects. For this reason, providing adequate pain management during this postoperative period is crucial and often involves a multimodal approach including oral, intravenous, intrathecal, and local analgesia [5].

Previous literature has demonstrated that local anesthetic wound infiltration during surgery is a simple, safe, and low‐cost technique for postoperative analgesia that can reduce systemic side effects associated with central neural blockades, the risk of anesthetic toxicity, analgesic requirements, and opioid‐related side effects [8]. While this technique has shown to be effective after minor surgical procedures, prior data examining the use of wound infiltration at the time of CD has been inconsistent.

In 2009, a meta‐analysis from the Cochrane Pregnancy and Childbirth Group's Trials Register found four studies (175 participants) that concluded the utilization of local anesthetic wound infiltration as post‐cesarean analgesia resulted in a 1.7 mg decrease in opioid consumption at 24 h [26]. In 2016, a study published in *The European Journal of Anaesthesiology* found 17 additional studies and conducted a systematic review and meta‐analysis, which concluded that while local anesthetic wound infiltration may reduce postoperative opioid consumption (decrease in 9.69 morphine equivalents), it may have only a minimal effect on pain scores and may not reduce opioid‐related side effects in women who had a CD [27]. However, the study was limited by the scarcity of studies using intrathecal morphine.

Our findings support previous evidence suggesting the improvement in pain score outcomes with local anesthetic wound infiltration in the first 48 h after surgery. This is consistent with results from a meta‐analysis including 1267 patients which found a statistically significant reduction in pain scores at rest and during movement with local anesthetic wound infiltration in first 24 h [27]. However, no statistically significant reduction in pain scores was noted at 48 h with movement or at rest, unlike in our study.

Previous literature has also demonstrated a reduction in cumulative morphine consumption in the immediate postoperative period. One meta‐analysis consisting of 512 patients demonstrated a reduced cumulative morphine consumption in the first 12, 24, and 48 h after surgery in patients receiving local anesthetic wound infiltration [28]. Similarly, a meta‐analysis by Adesope et al noted a decrease in opioid consumption at 24 h in patients with local wound infiltration [27]. However, a subgroup analysis noted that this statistical significance was only found in patients who received local wound infiltration without intrathecal morphine. In the two studies with intrathecal morphine, opioid morphine consumption at 24 h was similar in the groups with wound infiltration versus without. In contrast, our results demonstrate a significant reduction in morphine consumption in patients receiving wound infiltration in the presence of intrathecal opioids at 48 h. Additionally, subgroup analysis between single wound infiltration and continuous wound catheter infusion continues to note decreased morphine consumption. Our findings are consistent with the understanding that postoperative pain is multifactorial and different mainstays of treatment may be needed to address pain: wound infiltration can block both somatic and visceral afferents, whereas intrathecal morphine directly binds to opiate receptors in the subarachnoid space [27, 29, 30]. Use of a simple technique, such as a single wound infiltration, can be used to decrease opioid consumption and pain scores at time of CD.

Prior studies have demonstrated that when combined with IT opioids, there was no benefit in pain control after CD with the addition of quadratus lumborum block or TAP block. This suggests the significant role of IT opioids in adequately managing postoperative pain, whereas local anesthesia may be an unnecessary adjunct [31]. However, literature from colorectal surgeries have demonstrated that use of IT opioid with TAP block was associated with reduced early postoperative and total inpatient opioid use, days to functional recovery, and length of stay among patients undergoing abdominal surgery. To obtain a better understanding of the effects of local wound infiltration, only studies using IT opioids were selected for analysis [32]. An additional study evaluated the static pain score at 24 h postdelivery and found minimally clinically significant analgesic benefits when continuous wound infiltration was added to their standard multimodal analgesic protocol of intrathecal opioids (fentanyl 15 µg and morphine 100 µg), acetaminophen, and NSAIDs postdelivery [33]. The inclusion criteria in this study, however, only included women scheduled for a CD during the quality improvement period and excluded based on personal, medical, or surgical criteria. This study differs from our study given lack of control group, but it does provide insight into the addition of continuous wound infiltration to intrathecal opioids.

### 5.3 Strengths and limitations

The main strength of this systematic review is inclusion of only RCTs published to date comparing use of local wound infiltration anesthesia with intrathecal opioids at time of CD compared with standard of care. This review also included studies from diverse geographic locations and patient populations. The majority of studies included pain scores using standardized pain scales, such as visual analog scale (VAS) and numerical rating scale (NRS).

This study has some notable limitations inherent as a meta‐analysis. Our primary and secondary outcomes of pain scores were inadequately assessed at different time points for all comparative groups due to insufficient available data. Techniques for wound infiltration differed between RCTs with use of either single wound infiltration injection or continuous catheter placement, but both methods are included to provide information on the effectiveness of either technique. Since each study adhered to different local anesthetic and intrathecal dosing regimens, this variability can significantly impact the postoperative pain and thus confound the results. Including both short‐acting and long‐acting intrathecal opioids such as fentanyl, sufentanil, and morphine in the analysis can limit the consistency in the analysis, but they are included due to limited available data. Short‐acting opioids may enhance the effect of intrathecal local anesthetics and not significantly contribute to postoperative analgesia, whereas long‐acting opioids can provide more sustained relief. There are a few other sources of variability that contribute to possible bias in evaluation of differences including the following: type of local anesthetic used (ropivacaine, levobupivacaine, or bupivacaine), procedure (single injection or continuous catheter infiltration), and location of the infiltration (subfascial, suprafascial, and skin). These differences are displayed in Tables 6 and 8. Due to variability among studies and the small number of participants, ropivacaine, bupivacaine, and levobupivacaine could not be directly compared. However, notable differences exist—particularly between ropivacaine and bupivacaine—with respect to cost, availability, onset and duration of action, and potential toxicity. Future studies should specifically evaluate these agents in a comparative manner to better inform clinical practice protocols for amount and timing of additional pain medications, such as NSAIDS and acetaminophen, differed between studies as well, furthering concerns for study heterogeneity. The doses and timings between the different studies are shown in Table 7. Subgroup analyses comparing primary and secondary outcomes between different wound infiltration techniques could not be performed due to risk of confounding bias given differences in patient populations. Majority of studies included in the meta‐analysis utilized a single wound infiltration injected into the subfascial space using either ropivacaine, levobupivacaine, or bupivacaine, with improvement in both pain scores at 48 h and decrease in opioid consumption noted.

**TABLE 8:** Intraoperative characteristics.

Name, year	Skin incision	Repeated cesarean *N* (%)	Duration of surgery (min)
Pavy, 1994 [17]	NR	NR	NR
Ducarme, 2012 [18]	Joel‐Cohen, Pfannenstiel, or subumbilical midline laparotomy	46 (85.2) (I) 35 (79.5) (C)	44.6 ± 14.1(I) 46.1 ± 12.8(C)
Niklasson, 2012 [19]	NR	NR	NR
Jolly, 2015 [20]	Misgav Ladach: 68 (100%) (I) 68(C) (100%)	24 (71) (I) 22 (65) (C)	NR
Larsen, 2015 [21]	Pfannenstiel: 87 (100%) (I) 87 (100%) (C)	NR	NR
Prabhu, 2018 [22]	Pfannenstiel: 79 (100%) (I) 79 (100%) (C)	24 (61.5) (I) 26 (62.5) (C)	72 (66–90) (I) 72 (66–84) (C)
Rosetti, 2021 [23]	Pfannenstiel: 69 (100%) (I) 69 (100%) (C)	15 (42.9) (I) 16 (47.1) (C)	69 (I) 69 (C)
Gomez‐Rios, 2022 [24]	Pfannenstiel: 70 (100%) (I) 70 (100%) (C)	6 (18.2) (I) 7 (18.9) (C)	120 (100–135) (I) 120 (100–130) (C)
Totals	Pfannenstiel: 305 (I) 305 (C) Misgay Ladach: 68 (I) 68 (C)	115/197 (58) (I) 106/201 (52) (C)	

*Note*: Data are reported as number (percentage), mean ± standard deviation, or median (interquartile range).

Abbreviations: C, control; I, Intervention.

Another limitation of this study is that many of the primary studies had incomplete blinding or inadequate outcome reporting, which may introduce bias in subjective pain assessments. Additionally, most of the included studies involved elective cesarean deliveries in low‐risk populations. Therefore, the findings may not be generalizable to urgent or emergent cesarean deliveries, patients with obesity, or women with high opioid requirements. Many studies limited their inclusion criteria, excluding these populations; however, future research should aim to evaluate the effectiveness of this method in more diverse clinical settings.

A few studies were not able to be included for analysis of primary and secondary outcomes because their data were displayed with medians instead of mean and standard deviation. When using the online converter mentioned in the methods, the data appeared too skewed to be appropriate for use in analysis. The limited study data available for the primary and secondary outcomes and inherent variability between studies in factors that influence the postoperative pain, the findings may be biased. In addition, the original protocol included opioid consumption at 24 h as a primary outcome, but the statistics in the included studies reported these data in the median which resulted in the data being too skewed for use when converted to the mean.

Additionally, we were unable to address long‐term outcomes using these data including chronic pain. Patients included in these trials consisted of low‐risk patients for developing postoperative pain and opioid dependency. Further studies are needed examining the use of local wound infiltration at time of CD in reducing the need for opioid consumption in a high‐risk population. Although the clinical significance in the pain score difference between groups is small, the clinical significance for the opioid consumption is notable for the current opioid crisis we face in the country today. This study contributes to data needed to investigate the methods to reduce opioid consumption. Prior studies have demonstrated that recovery of physical activity after cesarean delivery is tightly correlated with pain. Therefore, improvements in pain score in the first 48 h may facilitate earlier ambulation and improved postoperative [34].

## 6 CONCLUSIONS

Our findings demonstrate that use of a single about 20–80 cc local wound infiltration, placed in the subfascial space with ropivacaine, levobupivacaine, or bupivacaine, at time of CD can significantly reduce pain scores 48 h postoperative period with movement and opioid consumption in the immediate postoperative period without increasing risk of adverse events. A multimodal approach to managing pain in patients who deliver via CD should be considered to improve postoperative outcomes.

## CONFLICT OF INTEREST STATEMENT

The authors declare no conflicts of interest.

## FUNDING INFORMATION

The authors received no specific funding for this work.

## References

[1] Weiser, T. G. , B. H. Alex , M. George , R. L. Stuart , M. E. Micaela , U. L. Tarsicio , F. Rui , et al. 2015. “Estimate of the Global Volume of Surgery in 2012: An Assessment Supporting Improved Health Outcomes.” Lancet 385: S11. 10.1016/S0140-6736(15)60806-6 26313057

[2] Garimella, V. and C. Cellini . 2013. “Postoperative Pain Control.” Clinics in Colon and Rectal Surgery 26(3): 191–6. 24436674 10.1055/s-0033-1351138 PMC3747287

[3] Laurent, B. , G. Lim , P. Sultan , A. S. Habib , R. Landau , M. Zakowski , M. Tiouririne , S. Bhambhani , and B. Carvalho . 2021. “Society for Obstetric Anesthesia and Perinatology: Consensus Statement and Recommendations for Enhanced Recovery After Cesarean.” Anesthesia & Analgesia 132, no. 5: 1362–77. 33177330 10.1213/ANE.0000000000005257

[4] Sultan, P. , N. Sharawi , L. Blake , A. S. Habib , K. F. Brookfield , and B. Carvalho . 2021. “Impact of Enhanced Recovery After Cesarean Delivery on Maternal Outcomes: A Systematic Review and Meta‐Analysis.” Anaesthesia Critical Care & Pain Medicine 40(5): 100935. 10.1016/j.accpm.2021.100935 34390864

[5] Eisenach, C. , P. H. Pan , R. Smiley , P. Lavand'homme , R. Landau , and T. T. Houle . 2008. “Severity of Acute Pain After Childbirth, but Not Type of Delivery, Predicts Persistent Pain and Postpartum Depression.” Journal of the International Association for the Study of Pain 140(1): 87–94. 10.1016/j.pain.2008.07.011 PMC2605246 18818022

[6] Hedderson, M. , D. Lee , E. Hunt , K. Lee , F. Xu , A. Mustille , J. Galin , C. Campbell , C. Quesenberry , V. Reyes , M. Huang , B. Nicol , S. Paulson , and V. Liu . 2019. “Enhanced Recovery After Surgery to Change Process Measures and Reduce Opioid Use After Cesarean Delivery: A Quality Improvement Initiative.” Obstetrics and Gynecology 134(3): 511–9. 31403591 10.1097/AOG.0000000000003406 PMC7282661

[7] Scott, N. B. 2010. “Wound Infiltration for Surgery.” Anaesthesia 65: 67–75. 20377548 10.1111/j.1365-2044.2010.06241.x

[8] Stamenkovic, D. M. , M. Bezmarevic , S. Bojic , D. Unic‐Stojanovic , D. Stojkovic , D. Z. Slavkovic , V. Bancevic , N. Maric , and M. Karanikolas . 2021. “Updates on Wound Infiltration Use for Postoperative Pain Management: A Narrative Review.” Journal of Clinical Medicine 10(20): 4659. 34682777 10.3390/jcm10204659 PMC8537195

[9] Rawal, N. 2023. “Intrathecal Opioids for the Management of Post‐Operative Pain.” Best Practice & Research Clinical Anaesthesiology 37(2): 123–32 37321761 10.1016/j.bpa.2023.01.001

[10] Grape, S. , K. El‐Boghdadly , and E. Albrecht . 2023. “Management of Adverse Effects of Intrathecal Opioids in Acute Pain.” Best Practice & Research Clinical Anaesthesiology 37, 2: 199–207. 37321767 10.1016/j.bpa.2023.02.002

[11] Page, M. J. , J. E. McKenzie , P. M. Bossuyt , I. Boutron , T. C. Hoffmann , C. D. Mulrow , L. Shamseer , et al. 2021. “The PRISMA 2020 Statement: An Updated Guideline for Reporting Systematic Reviews.” BMJ. 372: n71. 10.1136/bmj.n71. 33782057 PMC8005924

[12] Bezerra, C. T. , A. J. Grande , V. K. Galvão , D. Santos , Á. N. Atallah , and V. Silva . 2022. “Assessment of the Strength of Recommendation and Quality of Evidence: GRADE Checklist. A Descriptive Study.” Sao Paulo Medical Journal 140(6): 829–36. 36102459 10.1590/1516-3180.2022.0043.R1.07042022 PMC9671561

[13] Mean Variance Estimation . Accessed 28 May 2024. www.math.hkbu.edu.hk/~tongt/papers/median2mean.html.

[14] “Morphine Milligram Equivalents (MME) Calculator.” *MDCalc*. Accessed 28 May 2024. www.mdcalc.com/calc/10170/morphine‐milligram‐equivalents‐mme‐calculator.

[15] Review Manager (RevMan) (2014) Version 5.3. Copenhagen: The Nordic Cochrane Centre, The Cochrane Collaboration.

[16] DerSimonian R. and N. M. Laird . 1986. “Meta‐Analysis in Clinical Trials.” Controlled Clinical Trials 7: 177–88 3802833 10.1016/0197-2456(86)90046-2

[17] Pavy, T. , D. Gambling , P. Kliffer , A. Munro , P. M. Merrick , and J. Douglas . 1994. “Effect of Preoperative Skin Infiltration With 0.5% Bupivacaine on Postoperative Pain Following Cesarean Section Under Spinal Anesthesia.” International Journal of Obstetric Anesthesia 3(4): 199–202. 15636950 10.1016/0959-289x(94)90068-x

[18] Ducarme, G. , S. Sillou , A. Wernet , C. Davitian , O. Poujade , P.‐F. Ceccaldi , B. Bougeois , and L. Luton . 2012. “Intérêt de L'Instillation PariéTale Unique de Ropivacaïne Dans la prévention des Douleurs après césarienne. [Single‐shot Ropivacaine Wound Infiltration During Cesarean Section for Postoperative Pain Relief].” Gynécologie Obstétrique & Fertilité 40(1): 10–3. 10.1016/j.gyobfe.2011.07.035 22024157

[19] Niklasson, B. , A. Börjesson , U. B. Carmnes , M. Segerdahl , S. G. Ohman , and A. Blanck . 2012. “Intraoperative Injection of Bupivacaine‐Adrenaline Close to the Fascia Reduces Morphine Requirements After Cesarean Section: A Randomized Controlled Trial.” Acta Obstetricia et Gynecologica Scandinavica 91(12): 1433–9. 22686512 10.1111/j.1600-0412.2012.01480.x

[20] Jolly, C. , F. Jathières , H. Keïta , E. Jaouen , B. Guyot , and A. Torre . 2015. “Cesarean Analgesia Using Levobupivacaine Continuous Wound Infiltration: A Randomized Trial.” European Journal of Obstetrics & Gynecology and Reproductive Biology 194: 125–30. 26366789 10.1016/j.ejogrb.2015.08.023

[21] Larsen, K. R. , B. B. Kristensen , M. A. Rasmussen , Y. H. Rasmussen , T. Weber , B. Kristensen , and H. Kehlet . 2015. “Effect of High‐Volume Systematic Local Infiltration Analgesia in Caesarean Section: A Randomised, Placebo‐Controlled Trial.” Acta Anaesthesiologica Scandinavica 59(5): 632–9. 25786811 10.1111/aas.12509

[22] Prabhu, M. , M. A. Clapp , E. McQuaid‐Hanson , S. Ona , T. OʼDonnell , K. James , B. T. Bateman , B. J. Wylie , and W. H. Barth Jr . 2018. “Liposomal Bupivacaine Block at the Time of Cesarean Delivery to Decrease Postoperative Pain: A Randomized Controlled Trial.” Obstetrics and Gynecology 132(1): 70–8. 29889750 10.1097/AOG.0000000000002649

[23] Rosetti, J. , J. Francotte , E. Noel , P. Drakopoulos , N. Rabbachin , and M. de Brucker . 2021. “Continuous Ropivacaine Subfascial Wound Infusion After Cesarean Delivery in Pain Management: A Prospective Randomized Controlled Double‐Blind Study.” International Journal of Gynaecology and Obstetrics 154(1): 79–84. 33330983 10.1002/ijgo.13544

[24] Gómez‐Ríos, M. Á. , P. Codesido‐Barreiro , C. Seco‐Vilariño , M. Calvín‐Lamas , F. Curt‐Nuño , L. Nieto‐Serradilla , M. T. Rabuñal‐Álvarez , et al. 2022. “Wound Infusion of 0.35% Levobupivacaine Reduces Mechanical Secondary Hyperalgesia and Opioid Consumption After Cesarean Delivery: A Prospective, Randomized, Triple‐Blind, Placebo‐Controlled Trial.” Anesthesia & Analgesia 134(4): 791–801. 10.1213/ANE.0000000000005917. 35086112

[25] Gamez, B. H. and A. S. Habib . 2018. “Predicting Severity of Acute Pain After Cesarean Delivery: A Narrative Review.” Anesthesia & Analgesia 126(5): 1606–14. 29210789 10.1213/ANE.0000000000002658

[26] Bamigboye, A. A. and G. J. Hofmeyr . 2009. “Local Anaesthetic Wound Infiltration and Abdominal Nerves Block During Caesarean Section for Postoperative Pain Relief.” Cochrane Database Systematic Reviews Cd006954. 10.1002/14651858.CD006954.pub2 PMC13389773 19588413

[27] Adesope, O. , U. Ituk , and A. S. Habib . 2016. “Local Anaesthetic Wound Infiltration for Postcaesarean Section Analgesia: A Systematic Review and Meta‐Analysis.” European Journal of Anaesthesiology 33(10): 731–42. 27259092 10.1097/EJA.0000000000000462

[28] Li, X. , M. Zhou , X. Shi , H. Yang , Y. Li , J. Li , M. Yang , and H. Yuan . 2015. “Local Anaesthetic Wound Infiltration Used for Caesarean Section Pain Relief: A Meta‐Analysis.” International Journal of Clinical and Experimental Medicine 8(6): 10213–24. 26309720 PMC4538135

[29] Thomas, D. , S. Panneerselvam , P. Kundra , P. Rudingwa , R. K. Sivakumar , and G. Dorairajan . 2019. “Continuous Wound Infiltration of Bupivacaine at Two Different Anatomical Planes for Caesarean Analgesia—A Randomised Clinical Trial.” Indian Journal of Anaesthesia 63(6): 437–43. 31263294 10.4103/ija.IJA_745_18 PMC6573051

[30] Cummings, A. , B. D. Orgill , and B. M. Fitzgerald . 2024. Intrathecal Morphine. Treasure Island, FL: StatPearls Publishing. 29763055

[31] Sultan, P. , S. Patel , S. Jadin , B. Carvalho , and S. Halpern . 2020. “Transversus Abdominis Plane Block Compared With Wound Infiltration for Postoperative Analgesia Following Cesarean Delivery: A Systematic Review and Network Meta‐Analysis.” Canadian Journal of Anaesthesia 67: 1710e27 33089415 10.1007/s12630-020-01839-6

[32] Burchard, P. R. , A. D. Melucci , O. Lynch , A. Loria , Y. A. Dave , M. Strawderman , L. O. Schoeniger , E. Galka , J. Moalem , and D. C. Linehan . 2022. “Intrathecal Morphine and Effect on Opioid Consumption and Functional Recovery After Pancreaticoduodenectomy.” Journal of American College of Surgeons 235(3): 392–400. 10.1097/XCS.0000000000000261. PMC9371061 35758927

[33] Fowler, C. , E. Stockert , D. Hoang , N. Guo , E. Riley , P. Sultan , and B. Carvalho . 2024. “Continuous Wound Infusion Catheter as Part of a Multimodal Analgesia Regimen for Post‐Caesarean Delivery Pain: A Quality Improvement Impact Study.” BJA Open 9, 100242. 38179106 10.1016/j.bjao.2023.100242 PMC10761342

[34] Sharpe, E. E. , J. L. Booth , T. T. Houle , P. H. Pan , L. C. Harris , C. A. Aschenbrenner , and J. C. Eisenach . 2019. “Recovery of Physical Activity After Cesarean Delivery and Its Relationship With Pain.” Pain. 160(10): 2350–7. 10.1097/j.pain.0000000000001628 31145215 PMC6768712

